# Bacterial Surface Appendages Strongly Impact Nanomechanical and Electrokinetic Properties of *Escherichia coli* Cells Subjected to Osmotic Stress

**DOI:** 10.1371/journal.pone.0020066

**Published:** 2011-05-31

**Authors:** Grégory Francius, Pavel Polyakov, Jenny Merlin, Yumiko Abe, Jean-Marc Ghigo, Christophe Merlin, Christophe Beloin, Jérôme F. L. Duval

**Affiliations:** 1 Laboratoire de Chimie Physique et Microbiologie pour l'Environnement, Nancy Université, CNRS UMR7564, Villers-lès-Nancy, France; 2 Laboratoire Environnement et Minéralurgie, Nancy Université, CNRS UMR7569, Vandoeuvre-lès-Nancy, France; 3 Institut Pasteur, Unité de Génétique des Biofilms, Paris, France; 4 CNRS URA 2172, Paris, France; Clarkson University, United States of America

## Abstract

The physicochemical properties and dynamics of bacterial envelope, play a major role in bacterial activity. In this study, the morphological, nanomechanical and electrohydrodynamic properties of *Escherichia coli* K-12 mutant cells were thoroughly investigated as a function of bulk medium ionic strength using atomic force microscopy (AFM) and electrokinetics (electrophoresis). Bacteria were differing according to genetic alterations controlling the production of different surface appendages (short and rigid Ag43 adhesins, longer and more flexible type 1 *fimbriae* and F *pilus*). From the analysis of the spatially resolved force curves, it is shown that cells elasticity and turgor pressure are not only depending on bulk salt concentration but also on the presence/absence and nature of surface appendage. In 1 mM KNO_3_, cells without appendages or cells surrounded by Ag43 exhibit large Young moduli and turgor pressures (∼700–900 kPa and ∼100–300 kPa respectively). Under similar ionic strength condition, a dramatic ∼50% to ∼70% decrease of these nanomechanical parameters was evidenced for cells with appendages. Qualitatively, such dependence of nanomechanical behavior on surface organization remains when increasing medium salt content to 100 mM, even though, quantitatively, differences are marked to a much smaller extent. Additionally, for a given surface appendage, the magnitude of the nanomechanical parameters decreases significantly when increasing bulk salt concentration. This effect is ascribed to a bacterial exoosmotic water loss resulting in a combined contraction of bacterial cytoplasm together with an electrostatically-driven shrinkage of the surface appendages. The former process is demonstrated upon AFM analysis, while the latter, inaccessible upon AFM imaging, is inferred from electrophoretic data interpreted according to advanced soft particle electrokinetic theory. Altogether, AFM and electrokinetic results clearly demonstrate the intimate relationship between structure/flexibility and charge of bacterial envelope and propensity of bacterium and surface appendages to contract under hypertonic conditions.

## Introduction

The reactivity of bacteria with respect to their close environment is largely connected to biochemical and physicochemical properties defining the microorganism interphase, *i.e.* the cell wall, that spatially separates the bacteria from the outer medium [1]. For gram-negative bacteria, the cell wall consists of an outer membrane, which contains lipopolysaccharides (LPS) and surrounds a gel-like periplasm with a thin peptidoglycan layer [1], [2], [3], [4]. These cell wall components are separated from the cytoplasm by the inner membrane predominantly composed of phospholipids with embedded proteins. Despite their deceptively simple organization, gram-negative nude cell wall are involved in a vast array of complex cellular processes that serve key biological functions, *e.g.* ion channel conductance [5], cell signaling [6], cell growth or cell division [4], [7]. Additionally, these constituents are known to be essential in maintaining cellular shape [4] and in resisting internal Turgor pressure. For numerous bacterial systems, the cell wall is further decorated by surface layer organizations of the type *pili*, *fimbriae*, *curli*, exopolysaccharides (EPS) or flagella. These so-called surface appendages may protude several hundreds of nanometers from the anchoring cell wall. It is now well-reported that such bacterial surface ultrastructures are involved in numerous physical and biological interfacial processes, *e.g.* plasmid transfer through conjugation [8], adherence to materials or host cell surfaces [9], cell-cell interactions [10], biofilm formation [11], [12], [13], [14], [15], motility [16], [17], [18] and pathogenicity [19], [20], [21].

In the course of cellular growth and division, or in response to osmotic changes within the neighboring environment, cell wall undergoes morphological constraints affecting in some cases their integrity. For cell walls to appropriately counteract the inner Turgor pressure and allow efficient bacterial growth and division, it is necessary that their mechanical properties reflect the behavior of both stiff and ductile materials. Previous work demonstrated that mechanical properties of bacteria, including cell wall elasticity, change significantly as a result of forces acting on bacterial structure as it is the case during cell growth and division [22], [23], or during adhesion and infection processes [9], [24], [25]. In view of these elements, a fundamental comprehension of the physiological processes and reactivity of bacterial cells necessarily requires, besides the underlying details on gene expression [26], [27], [28], accurate measurement and interpretation of their mechanical properties [22], [29], [30], [31], [32] in relation with envelope structure that includes not only cell wall but also surface appendages.

In the past decades, much progress has been made in understanding the mechanical and more generally the physico-chemical properties of microorganisms [33], [34]. However, due to the small size of the cells, these properties remain difficult to address at a nanometric level. In this context, atomic force microscopy (AFM) has emerged as a valuable and powerful tool [35] for studying nanomechanical characteristics of living cells [36], [37], [38]. Other practical applications of AFM includes the imaging of cell ultrastructures such as *pili*, *fimbriae* [14], [39], [40] or the elucidation of the impact of antibacterial molecules [38], [41], [42] on genetically modified bacteria [37], [43]. A major advantage of AFM is that it allows measurements of surface nanostructure in aqueous media of controlled composition, which makes it ideal for analyzing cell wall response to osmotic stress. Although the structure and possible chemical make-up of Gram-negative cell wall may be now accurately identified, AFM studies on mechanical properties of individual living cells in aqueous medium as a function of salt concentration remain scarce. In particular, unraveling the respective contribution of long or short external structures in governing bacterial envelope elasticity, Turgor pressure and stretching modulus (*i.e.* bacterial surface tension) remain an issue. Also, the impact of cell wall ultrastructure reorganization following swelling/stretching processes on nanomechanical properties of the bacterial envelope as a whole, has deserved too little attention despite the fundamental importance of these phenomena in governing bacterial reactivity under hypotonic/hypertonic stress conditions.

In this study, we report a systematic investigation of the ionic strength dependent-nanomechanical properties of *E. coli* K-12 mutant strains which selectively express (or not) surface appendages such as type 1 *fimbriae*, F conjugative *pilus* or autotransported adhesin antigen 43, known to be involved in biofilm formation and/or bacterial pathogenicity [15], [20], [44], [45], [46], [47], [48] (see Materials & Methods for a full description of these surface structures). Analysis of the AFM force-indentation curves yields the Young moduli, internal Turgor pressures and stretching moduli of the bacteria of interest as a function of medium ionic strength. It further allows evaluating not only how nanomechanics is impacted by envelope structure but also addressing the changes operated on this structure in hypertonic stress conditions. The AFM study is complemented by electrokinetic measurements which, upon modeling on the basis of theory for permeable (soft) bioparticles, highlight the relation existing between density of charges carried by the surface appendage, its propensity to swell upon lowering medium salt content and its intrinsic elasticity/rigidity as determined independently by AFM. Overall, this work quantitatively underlines the intertwined relationships between nature of bacterial envelope structure, their dynamic features (swelling), and physico-chemical properties expressed in terms of nanomechanical, electrostatic and hydrodynamic (permeability) characteristics.

## Materials and Methods

### Bacterial strains

**Figure 1:**
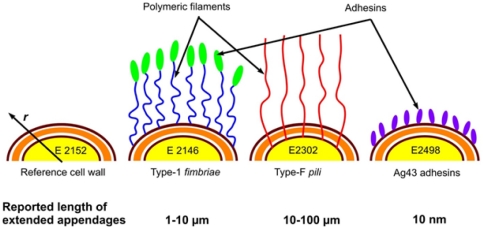
Spatial organization of the external structures for the various *E coli* strains investigated. *Pili* and *fimbriae* are structures with reported total lengths of about ∼10-100 µm (E2302) and ∼1-10 µm (E2146), respectively, while antigen Ag43 protein layer is a short and rigid structure ∼10 nm thick (E2498).

The *E. coli* K-12 strains used in this study are listed in **Table 1** and **Table 2** where relevant information on their respective construction, antibiotic resistance, genotype and surface appendages expression can be found. These isogenic strains were constructed from *Escherichia coli* MG1655 (*E. coli* genetic stock center CGSC#6300) by transformation and λ red linear DNA gene inactivation method using the pKOBEG plasmid [49], [50], followed by P1*vir* transduction into a fresh *E. coli* background when possible. Alternatively, strains were constructed by P1*vir* transduction of previously constructed and characterized mutation or insertion. All strains used in this study contain the *gfpmut3* gene linked to the *bla* ampicillin resistance gene (*amp* ^R^, 100 µg/ml) that makes them fluorescent, and a deletion of the *fliE* to *fliR* genes replaced by the *cat* chloramphenicol resistance gene (*cm* ^R^, 25 µg/ml), which ensures the absence of flagella. Absence of flagella was verified by absence of motility using motility assay on low agar motility plates (data not shown). Our reference strain (E2152) has been constructed by creation and P1*vir* transduction of mutations previously shown to i/abolish type 1 *fimbriae* production (deletion of the type 1 *fimbriae* encoding operon, Δ*fimA-H*: *zeo, zeo* ^R^ 50 µg/ml), or ii/abolish adhesin Ag43 production (deletion of the *flu* gene, Δ*flu*: *km*, *km* ^R^ 100 µg/ml or Δ*flu*: *zeo*, *zeo* ^R^ 50 µg/ml). Primers used to construct the Δ*fliE-R*: *cat*, Δ*flu*: *km*, Δ*flu*: *zeo* and the Δ*fimA-H*: *zeo* deletions are listed in **Table 1**. The strain E2152, devoid of these 3 surface appendages, was selected as a reference for comparing AFM data and electrokinetic results with those obtained for the strains E2146, E2498 and E2302 which constitutively produces the external ultrastructure type 1 *fimbriae*, the Ag43 protein and the type-F pili, respectively (**Fig 1**). Constitutive expression of the Ag43 protein was previously obtained by placing a constitutive promoter in front of the *flu* gene [12] whereas constitutive expression of the type 1 *fimbriae* was previously obtained by placing a constitutive promoter in front of the *fimA-H* operon [51]. Production of the F *pili* was obtained by introducing the F'tet plasmid into the reference strain E2152 by conjugation, creating E2302. All constructions were verified by PCR. The E2152, E2146, E2302 and E2498 strains deleted for- or selectively expressing- the three different appendages listed above were phenotypically checked before proceeding with the experiments reported in this study. All three strains displayed phenotypes in line with previous description [12], [47], [52]. In details, we systematically verified that i/absence and constitutive production of type 1 *fimbriae* were respectively verified by deficiency in- or enhancement of- yeast agglutination and biofilm formation; ii/absence and constitutive production of the Ag43 adhesin were respectively verified by deficiency in- or enhancement of- autoaggregation as well as by Western Blot immunodetection; iii/presence of the F *pili* was verified by sensitivity to phage M13 and increased biofilm formation (Table S1).

**Table 1:** Genetic and phenotypic characteristics of the studied bacteria.

Strain or plasmid	Relevant genotypic and phenotypic characteristics	Source or reference
**Strains**		
MG1655*gfp*	MG1655_λatt*amp_gfp*, Amp^R^GFP+	[12]
MG1655Δ*fli*	Δ*fliE-R*: *cat*, Cm^R^deletion of *fliE* to *fliR* genes by λ-red recombinationno flagella	This study
MG1655Δ*fim*	Δ*fimAICDFGH*: *zeo*, Zeo^R^deletion of *fimA* to *fimH* genes by λ-red recombinationno type 1 fimbriae	This study
MG1655Δ*flu*: *km*	Δ*flu*: *km*, Km^R^deletion of the *flu* gene by λ-red recombinationno Ag43 protein	This study
MG1655Δ*flu*: *zeo*	Δ*flu*: *zeo*, Zeo^R^deletion of the *flu* gene by λ-red recombinationno Ag43 protein	This study
MG1655*km*PcL*fim*	*fimAICDFGH* operon placed under the control of the *km*PcLrbs cassette λP_R_ promoter, Km^R^constitutive type 1 fimbriae	[51]
MG1655*km*PcL*flu*	*flu* gene placed under the control of the *km*PcLrbs cassette λP_R_ promoter, Km^R^constitutive Ag43	[12]
MG1655_F'tet	Strain containing the F conjugative plasmid, Tet^R^constitutive F pili	[47]
E2152	*gfp*_Δ*fliE-R*: *cat*_Δ*flu*: Km_Δ*fimAICDFGH*: *zeo*, Amp^R^, Cm^R^, Km^R^, Zeo^R^subsequent P1*vir* transduction of Δ*fliE-R*: *cat*, Δ*flu*: *km* and Δ*fimAICDFGH*: *zeo* in MG1655*gfp*GFP+, no flagella, no Ag43 protein, no type 1 fimbriae, no F factor	This study
E2146	*gfp*_Δ*fliE-R*: *cat*_Δ*flu*: *zeo*_*km*PcL*fim*, Amp^R^, Cm^R^, Km^R^, Zeo^R^subsequent P1*vir* transduction of Δ*fliE-R*: *cat*, Δ*flu*: *zeo* and *km*PcL*fim* in MG1655*gfp*GFP+, no flagella, no Ag43 protein, no F factor, constitutive type 1 fimbriae	This study
E2498	*gfp*_Δ*fliE-R*: *cat*_Δ*fimAICDFGH*: *zeo*_*km*PcL*flu*, Amp^R^, Cm^R^, Km^R^, Zeo^R^subsequent P1*vir* transduction of Δ*fliE-R*: *cat*, Δ*fimAICDFGH*: *zeo* and *km*PcL*flu* in MG1655*gfp*GFP+, no flagella, no type 1 fimbriae, no F factor, constitutive Ag43	This study
E2302	*gfp*_Δ*fliE-R*: *cat*_Δ*flu*: Km_Δ*fimAICDFGH*: *zeo*_F'tet, Amp^R^, Cm^R^, Km^R^, Zeo^R^, Tet^R^conjugation of F'tet within E2152GFP+, no flagella, no Ag43 protein, no type 1 fimbriae, F+	This study
		
	Plasmids	
pKOBEG	pSC101 ts (replicates at 30°C), araC, arabinose-inducible λredγβα operon, Cm^R^	[50]

Relevant genotype and phenotype characteristics of the various *E. coli* mutants selected for this study.

**Table 2:** Genetic sequences characteristics of the studied bacteria.

Target gene	Primer name	Sequence
		Primers used to generate deletion mutants in different *E. coli* strains
*fliE-R*	fliR.500-5	gttgattaatgatgagcttg
	fliE.500-3	tccaagacaactgactaact
	fliR.cat.L-3rev	CTGCGAAGTGATCTTCCGTCACAGGttccgtaacgtttatcatgttatc
	fliE.cat.L-5rev	GATGAGTGGCAGGGCGGGGCGTAAgttttgtaacctgttgttaattac
	fliR.ext-5	gaccaatagatattcatcaa
	fliE.ext-3	ctaattggtgtaaacttaccat
*flu*	flu.A1.500-5	CCCgAATTCTgCggTggACCggATATTTg
	flu.B1.500-3	atttccttgctgatatcttc
	flu.A2GB-L-3	gattttgagacacaacgtggctttCATcagcttttccttagattg
	flu.B2.GB-L-5	cttcacgaggcagacctcagcgccTAAcagaaccatcgcctctctgtg
	flu.ext-5	atacgctggtcagtgcgctc
	flu.ext-3	atcagtgacggtgaaatatc
	flu.zeo.L-5	TTCGTGGCCGAGGAGCAGGACTGAcagaaccatcgcctctctgtg
	flu.zeo.L-3	GTCAACACGTGCTCGGATCCAGAACATcagcttttccttagattg
*fimA-H*	fimA.500-5	GAGAAGAGGTTTGATTTAACT
	fimH.500-3	TCCAGCAACTGGTCAGCTGGTT
	fimA.zeo.L-3	GTCAACACGTGCTCGGATCCAGAAGCTGCTTTCCTTTCAAAAAA
	fimH.zeo.L-5	TTCGTGGCCGAGGAGCAGGACTGAGAAATCACAGGACATTGCTAATG
	fimA.ext-5	CTTGACCTTAATGAAGGTAG
	fimH.ext-3	TTTCGGCGTTTCGATTCTGGTGC

a Amp, ampicillin; Cm, chloramphenicol; Km, kanamycin; Tet, tetracycline; Zeo, zeocin.

Primers used to construct the Δ*fliE-R*: *cat*, Δ*flu*: *km* and the Δ*fimA-H*: *zeo* deletions are listed here.

### Biological functions and characteristics of the surface appendages examined in this study

Type 1 *fimbriae*, constitutively expressed in E2146 strain, are filaments consisting of proteins called fimbrilin anchored at the outer bacterial membrane. They exhibit a rod-shaped and helical structure of total contour length 1 to 10 µm and diameter 6 to 20 nm [53], [54]. Type 1 *fimbriae* play an important role in biofilm formation, bacterial pathogenicity and virulence [46] essentially because these structures render possible host cell colonization by bacteria. A protein called the FimH adhesin is located at the extremity of each *fimbriae*, and ensures adhesion to host cells following docking mechanism [55], [56]. Type-F *pili* or sex *pili* expressed in E2302 strain are associated to conjugation [57], [58]. They may be described as helically built tubules with a hollow core of 2 to 4 nm in diameter and a 10 to 100 µm long flexible filament [59] which consists of repeating units of pilin. These filamentous structures allow genetic transfer of plasmid genes [60], [61] but also infection by specific bacteriophages [62]. F pili have been shown to strongly promote adhesion to abiotic surface and biofilm formation [47]. Furthermore, we constructed a strain constitutively expressing Antigen 43 protein (E2498). The Ag43 protein is constituted by 2 different polypeptides and forms a ∼10 nm long structure known to be rigid [63]. This abundant outer membrane protein in *E. coli* belongs to the autotransporter family, promotes bacterial autoaggregation and biofilm formation, and is associated to urovirulence [20], [45], [64].

### Growth conditions

Bacteria were pre-grown overnight at 37°C under agitation (150 rpm) in M3B1 minimal medium supplemented with 0.4% glucose (M63B1glu) and with the appropriate antibiotic for the proper selection of the strain of interest. The following day, fresh M3B1glu medium was inoculated with the overnight culture to an OD_600_ of *c.a.* 0.05 and cultivated under the same conditions until the biomass reached an OD_600_ of 0.5-0.6.

Bacterial cells dedicated to AFM studies were extensively washed with 50 ml of 1 mM KNO_3_ (see details below) while cells dedicated to electrokinetic study were washed once in KNO_3_ salt medium prior to use: the cultures (10 mL aliquots) were centrifuged at 5000 g for 15 min at a temperature of 4°C, supernatants were then eliminated and the pellets were resuspended in an equal volume of KNO_3_ aqueous solution (of concentration 1 mM to 100 mM, range examined for electrophoresis analysis). Cells were centrifuged (5000 g, 4°C, 15 min) a second time and cell pellets were finally resuspended in KNO_3_ solution before use for electrokinetic experiments. The bacterial suspensions were adjusted in ionic strength upon addition of appropriate aliquots of 1 M KNO_3_ electrolyte solution. We verified that gentle centrifugation in the range 2000 g–5000 g did not impact the reported electrophoretic mobilities of our selected bacteria, recalling that electrophoresis is mostly sensitive to physico-chemical properties of their outermost peripheral region [65]. Concerning AFM experiments that probe internal parts of the bacteria *via* indentation experiments, we rather chose avoiding centrifugation of the bacteria in order to fully guarantee the absence of perturbation of their mechanical properties following change in physiology and/or ruptures of some of their surface appendages. Our experience is that, depending on the type of bacterial strain considered, centrifugation might lead to important surface structure modification as observed *e.g.* for *Pseudomonas fluorescens* (paper in preparation).

### Electrophoretic mobility measurements

Electrophoretic (electrokinetic) mobility (EPM) measurements were performed in a quartz suprasil cell at 24°C (Zetaphoremeter IV, CAD Instrumentations, Les essarts le Roi, France) and were determined from the reflection by bacteria of a laser beam tracked with a charge-coupled device camera. Using an image analysis software, recorded images were processed in real time to calculate the electrophoretic mobilities from the displacement (migration motion) of bacteria subjected to a constant direct-current electric field (800 V/m). Different cycles were recorded to carry out 100 measurements of bacterial mobility for a given KNO_3_ electrolyte concentration in the range 1 mM to 100 mM. Additionally, the reproducibility of the experiments was addressed by repeating the electrokinetic measurements with at least three different fresh bacterial dispersions.

### Electrophoretic mobility data processing for a soft bacterial particle

The ionic-strength dependence of the electrophoretic mobility data was quantitatively interpreted using the electrokinetic theory for cylindrical soft particles developed by Duval et *al*. and extensively described in [65]. For the sake of completeness, the modeling of the bacteria together with the basic physical elements of the aforementioned theory are briefly recalled below. The reader is referred to [65] for more complete details.

#### Theoretical representation of the bacterium/aqueous medium interphase

The bacteria of interest here may be assimilated to rod-like bioparticles of length *L* at the surface of which the cell wall and its appendages can be represented by a permeable, charged gel-like layer which exhibits a certain propensity for fluid (electroosmotic) flow penetration during the field-driven motion of the bacteria. Because of the presence of such permeable component, bacteria are recognized as paradigms of so-called soft (*i.e.* permeable) biocolloids [66], [67]. The bacteria are modeled here as soft particles comprised of (i) a hard-core of radius *a*, which is impermeable to electroosmotic flow and corresponds to the cytoplasmic part, and (ii) the permeable charged gel-like layer, of thickness *d*, located at the bacterial periphery around the cytoplasm. When modeling the electrokinetic behavior of the bacteria, it is necessary to account for the effect of interphasial swelling and corresponding chain heterogeneity on the local distribution of the material density within the bacterial soft envelope [65], [68]. This can be achieved by necessarily abandoning the conventional picture of the bacterial soft structure viewed as a homogeneous permeable (step-like) layer. Instead, we represent the bacterial interphase as a soft polyelectrolyte layer of which the density gradually decays from maximum value within the bulk gel-like layer to zero in the electrolyte solution. Without any detailed molecular information on the local distribution of the soft matter that surrounds the cytoplasm, the radial density distribution, *f*(*r*), of material that constitutes the permeable charged gel-like layer may be described by a function of the form [65]: 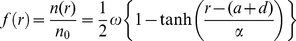
(1)where 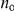 is the nominal density of soft material for a homogeneous gel layer distribution, *n*(*r*) is the radial dependence of the density of soft matter within the permeable charged gel-like layer, *r* the radial position according to the polar coordinate system taken at the center of the particle (see **Fig 1**). The characteristic parameter 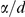 represents the degree of the diffuseness (or extension) of the interphase with the limit 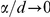 implying a step-like or homogeneous soft layer distribution. The dimensionless parameter ω is obtained from the required condition that the total quantity of gel material is constant upon variation of the interfacial diffuseness as subsumed in the ratio 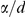. For highly hydrated soft surface layers and assuming uniform distribution of charges along a single chain, it was demonstrated that *f*(*r*) pertains to the distribution of the local friction exerted by the gel layer on electroosmotic flow and to the spatial dependence of the density of charges carried by the gel layer [68]:
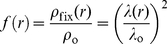
(2)where ρ_0_ and λ_0_ are the volume density of fixed charges and the hydrodynamic softness parameter within homogeneously distributed soft gel-like structure (limit 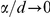), respectively. The quantity 1/*λ*_0_ defines the characteristic penetration length of electroosmotic flow within the permeable gel-like layer while *ρ*_fix_(*r*) and *λ*(*r*) denote the local volume charge density and hydrodynamic softness throughout the permeable part of the particle, respectively. A major advantage for introducing the quantity *α* in the representation of the bacterial soft component is that it allows for accounting situations where interfacial swelling takes place, with as a result an expansion of the gel layer, *i.e.* increase of its thickness [68], [69]. Based on the profile expressed by eq 1, the overall extension of the soft layer is about 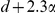 [68]. This extension is accompanied by an increased friction exerted by the outer edge of the bacterium soft layer on the electroosmotic flow, thereby reducing the magnitude of the mobility [68]. This feature, theoretically and experimentally addressed in previous publications [65], [68], [70], illustrates the utmost role played by polymer tails on hydrodynamic flow field developments, as qualitatively recognized long ago by Cohen-Stuart *et al.* [71], [72].

#### Electrokinetic model for a rod-like, diffuse soft particle

For a given electrolyte composition, the electrophoretic mobility 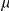, which is accessible by the experiment, is dependent on the various electrohydrodynamic quantities (*i.e.* the parameters *ρ*_0_ and *λ*_0_) pertaining to the bacterial cell wall or surface appendage assimilated to the gel-like layer, on its thickness, on the radius of the cytoplasm (core bacterial component) and also on the soft material distribution (homogeneous or diffuse) in the *r*-direction, *i.e.* on 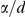. For a cylindrical particle, 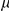 further depends on the orientation of the particle-long axis relatively to the direction of the applied electric field ([65] and see further references therein)
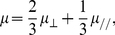
(3)where *µ*_⊥_ and *µ*_//_ are the electrophoretic mobilities of the cylindrical particle with its axis perpendicular and parallel to the applied electric field, respectively. The components *µ*_⊥_ and *µ*_//_ are obtained upon collocation-based numerical resolution [73] of the set of coupled Navier-Stokes, Poisson Boltzmann and continuity governing equations explicitly detailed in [65] for a particle of cylindrical geometry in an electrolyte of dielectric permittivity *ε*_0_ *ε*_r_ composed of i = 1,…,N different ionic species with valency *z*_i_, and bulk concentration *c*_i_^∞^. This theory does not suffer from any restriction on the magnitude of the particle core and shell sizes nor on the magnitude of the electrostatic potential within the shell layer. As such, it constitutes an extension of the approximate formalism by Ohshima [74], [75], [76] which is strictly applicable in the high ionic strength limit where electric double layer polarization phenomena are insignificant [70]. A review of successful applications of diffuse soft particle electrokinetic formalism, as briefly outlined above in the case of cylindrical particles, may be found in [70]. These latter studies illustrate the potentiality of electrokinetic methods for determining (i) electrohydrodynamic and swelling properties of (bio)particles like bacteria [77], yeast cells [78], viruses [79], erythrocytes [69] or colloidal microgels, as well as the (ii) changes in interfacial properties upon (bio)particle surface modification following exposure to biospecific molecules or enzymatic actions [69], [80].

### AFM measurements

AFM images and force-distance curves were recorded using an MFP3D-BIO instrument (Asylum Research Technology, Atomic Force F&E GmbH, Mannheim, Germany). Silicon nitride cantilevers of conical shape were purchased from Veeco (MLCT-AUNM, Veeco Instruments SAS, Dourdan, France), and their spring constants, denoted as *k*, were determined using the thermal calibration method [81], providing *k* values of ∼10.4±1.7 pN nm^-1^. Prior to experiment, the geometry of the tip was systematically controlled using a commercial grid for 3-D visualization (TGT1, NT-MTD Compagny, Moscow, Russia) and curvature of the tip in its extremity was found to lie in the range ∼20 to 50 nm. Experiments were performed in 1 mM and 100 mM potassium nitrate solution at pH∼6.6 and room temperature. Cells were electrostatically-immobilized onto polyethyleneimine (PEI)-coated glass slides according to a procedure detailed elsewhere [82]. Such method avoids the necessity to resort to chemical binders between substrate and bacterial sample, thus minimizing any chemical modification of bacterial cell wall/surface organization.

Glass slides were freshly prepared upon immersion in 0.2% PEI solution for 30 minutes, extensively rinsed with Milli-Q water, dried with nitrogen and stored in a sterile Petri dish. One mL of bacterial culture (OD_600 nm_∼0.5–0.6) was directly deposited onto the PEI-coated glass slide for 20 minutes and then the bacteria-coated surface was extensively rinsed 3 times with Milli-Q water. We emphasize that high molecular weight PEI (750 000 to 1 000 000 g/mol) was used for avoiding desorption and interaction with bacterial cell wall, and the absence of PEI contamination onto tip and samples was systematically verified. This important point was extensively addressed in our recent paper [83] dealing with the methodology for analysis of approach curves on *E. coli* 2152 and retraction curves on *Pseudomonas fluorescens* (construction of force volume images). In particular, it was verified that PEI did not alter the selectivity of (functionalized) tip with respect to given molecular compounds present on *Pseudomonas fluorescens* surface. In addition, the high molecular weight of PEI (polycationic polymer) used in this study forms a very stable monolayer strongly bond to (negatively charged) glass substrate due to important (attractive) electrostatic interaction. To the best of our knowledge, no PEI adsorption phenomenon has yet been reported in literature under the experimental conditions of interest here. Having in mind those elements, each bacterial sample was extensively rinsed with 50 ml of 1 mM KNO_3_ and then was immediately transferred into the AFM liquid cell with addition of 2 ml of KNO_3_ solution of adjusted concentration and pH∼6.6. Finally, we mention that bacteria immobilized onto the PEI-coated substrate were extensively rinsed with KNO_3_ in order to get rid of un-adhered free bacteria and possibly remaining residues from minimum growth medium.

### Nanomechanical AFM data processing

Mechanical properties were measured by recording a grid of 32-by-32 and 16-by-16 force curves obtained upon approach of the tip to the bacteria, using a maximum applied force of 4 to 5 nN in order to avoid sample damage. The bacterial spring constant, *k*_cell_, was determined from the slope of the linear portions of the raw deflection *versus* piezo displacement curves [84], [85], [86] according to:
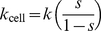
(4)with *s* the experimentally accessible slope of the compliance region reached for sufficiently large loading forces. The bacterial Young modulus was obtained by interpreting the non-linear regime in the force *versus* indentation curves according to Sneddon model [87]:
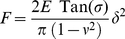
(5)where *F* is the loading force, δ the indentation depth, *E* the Young modulus, *ν* the Poisson coefficient, and *σ* the semi-top angle of the tip. Equation 5 refers to cases where compression and deformation of a soft planar interface is occasioned by a tip of conical geometry [88]. Recalling that the curvature radii of the probed bacteria are about two orders of magnitude larger than that of the tip we can consider a cone/plan contact for describing mechanical interaction. In addition, though eq 5 truly holds for indentation depths below the curvature radius of the AFM tip (*i.e.* in the first ∼50 nm of indentation), we verified that description of experimental data with eq 5 was very satisfactory for larger indentations. Young Moduli resulting from force curve interpretation over the whole non-linear indentation regime (including the first 50 nm where Sneddon model is accurate) or within the first 50 nm only, very well compare to magnitudes reported in literature for bacteria. Young moduli were determined for a piezodrive speed of 1000 nm/s (0.5 Hz), which ensures full relaxation of the bacterial surface structure upon successive measurements, as verified from additional experiments carried out at different speeds of piezodrive. This also fully justifies the use of the static physical model of Sneddon as adopted in our analysis. In addition, the model is valid for elastic surfaces on the premise that tip-surface adhesion is absent or insignificant. For the bacterial systems of interest here, this restriction is satisfied in view of the very low magnitude of the adhesion forces (<0.01 nN) measured upon retraction of the tip from the bacteria. The mathematical analysis was performed on 1024 measured force curves with an automatic Fortran C++ algorithm described elsewhere [37], [89] using *σ* = 35°, as inferred from tip geometry, and *ν* = 1/2. Under low ionic strength condition (1 mM salt concentration) and for loading forces typically lower than 0.1 nN, we systematically observed slight deviations of the indentations curves from those predicted according to eq 5. This feature will be analyzed in more details in a forthcoming publication. It is assigned to the effects of electric double layer repulsion and/or steric interaction between bacterial interphase and AFM tip [90], [91]. A detailed analysis of the force-separation distance curves (*i.e.* in the regime where there is no contact between tip and bacterium surface) reveals that the typical characteristic length of interaction between E2152 and tip in 1 mM electrolyte concentration is about 11 nm [83], which very well corresponds to the Debye layer thickness expected from theory at such salt content in solution. This supports the idea that the short range interactions between E2152 and tip, as measured by AFM, are predominantly governed by electrostatics, at least in case of the bare E2152 strain devoid of long surface appendage. For all strains, we scrupulously verified that ignoring the region where electrostatic/electrosteric interactions come into play for appropriately positioning the contact point between AFM and bacteria, leads to an error in the determination of Young moduli of about 1 to 4% in the worst cases, which remains within the error bar of AFM data (see details in **Figs S1-S2** and **Histogram S2** therein). In this study, ignoring the electrostatic/electrosteric part of the force curves for evaluating the nanomechanical parameters is justified in view of (i) the significant difference between spatial range of mechanical indentation and that where electrostatic forces are operative and (ii) the low value reported for the typical magnitude of electrostatic forces (∼0.1 nN, (38)) as compared to that of the loading forces (0–5 nN).

## Results

### Bacterial cell surface imaging

**Figure 2:**
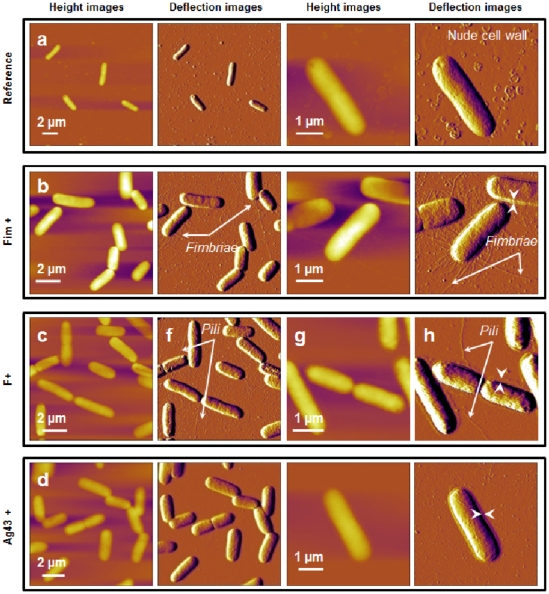
Bacterial morphology observed by AFM in air (contact mode). AFM height and deflection images (z-scale = 200 nm) recorded in air for *E coli* cells immobilized on a PEI-coated glass surface. a) E2152 b) E2146 c) E2302 d) E2498. The white arrows correspond to the thickness of a layer that surrounds the bacteria (see details in main text).

In order to correlate the presence or absence of surface appendages with their genetic and phenotypic characteristics, the four bacterial strains considered in this study were first imaged in air using AFM in contact mode. For that purpose, the bacteria-coated PEI surfaces were rinsed and gently dried with nitrogen. Additionally, while the resolution of the height images was limited due to the large curvature of the analyzed rod-shaped cells, deflection images were found to be significantly more sensitive to bacterial surface relief and morphological details, as shown in **Fig 2**.

The AFM images reported in **Fig 2** point out that E2152 bacterial strain can be assimilated to a 3 µm long rod-shaped cell of diameter 0.8 µm (**Fig 2a**). As expected, E2152 bacterial cell envelope did not exhibit any visible surface appendage. In the case of E2146 (**Fig 2b**) and E2302 (**Fig 2c**) strains, filamentous structures could be distinctly observed. Quantitative analyses of the images performed on a statistically representative number of cells provides appendage length for the E2146 strain in the range 0.2 to 10 µm with a diameter of about 10 nm, values that are in good agreement with those reported in literature for type-1 *fimbriae* [54], [92]. Additionally, it is found that E2146 cells are surrounded by a 100 nm to 200 nm thick layer, as materialized by the white arrows depicted in **Fig** **2b**. This layer, absent from the E2152 *E. coli* reference strain, probably consists of partially bent *fimbriae*. A heterogeneous envelope composed of 2 µm to 10 µm long filaments (∼10 nm in diameter) could be identified for the E2302 strain (**Fig 2c**). In view of the genetic construction of this strain, these filaments are identified as F-*pili*. Their dimension corresponds to the low size limit as reported in [93], [94] for such surface appendage. Similarly to E2146, E2302 further exhibits a 50 nm thick layer around the cell wall, the origin of which possibly stems from the presence of partially or completely stretched *F-pili* along the cell wall. No filamentous surface appendage could be evidenced for the E2498 strain (**Fig 2d**), in line with previous SEM studies indicating that Ag43 does not form recognizable extended surface structure [63]. Despite this, the presence of a peripheral 50 nm thin layer around E2498 cells could be unambiguously detected and it is believed that this layer reflects the presence of the expected Ag43 coating. The thickness of this protein layer is however larger than that reported in literature [45], [95]. Such discrepancy is possibly attributed to strong convolution of the image by particle geometry, recalling that curvature and edges of the cantilever tip of radius 20 to 50 nm are expected to contribute when imaging thin surface envelopes like Ag43 protein layer [96].

**Figure 3:**
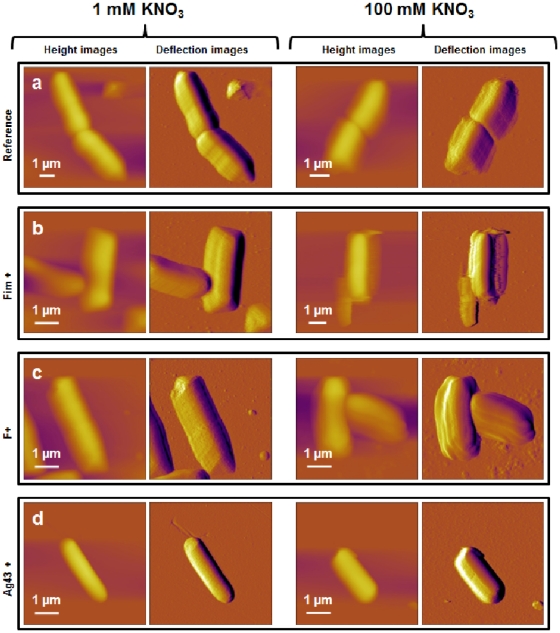
Bacterial morphology observed by AFM in aqueous medium (contact mode). AFM height and deflection images (*z*-scale = 2 µm and *d*-scale = 50 nm) recorded in 1 mM (left) and 100 mM (right) KNO_3_ aqueous solution for a single *E. coli* cell immobilized on a PEI-coated glass surface (*z*-scale = 2 µm and *d*-scale = 50 nm). a) E2152 b) E2146 c) E2302 d) E2498.

Following the previous findings, attempts were done to image the various bacteria in 1 mM and 100 mM KNO_3_ aqueous electrolyte so as to appreciate the dynamics of the above identified surface structures with varying salt medium content. Unfortunately, AFM technique used in contact mode turned to be inappropriate for imaging refined details pertaining to the bacterial surface ultrastructures of interest under wet conditions, as illustrated in **Fig 3**. This difficulty is imputed to the easy dislodging of the surface appendages by the AFM tip operating in fluid media according to contact mode, argument already mentioned by several authors [39], [97]. The required stress exerted by the tip on the bacterial structure for appropriate imaging of flexible *F-pili* or *fimbriae* filaments probably exceeds the forces responsible for their anchoring to the cell membrane and/or leads there to spatial displacement, thus rendering inefficient any surface scan for unraveling morphological details. Despite this limitation, images of bacteria as depicted in **Fig 3** allow for retrieving quantitative morphological information for the bacteria as a whole, in particular their propensity to swell or shrink when changing salt concentration.

The E2152 reference strain may be assimilated to a 2.9±0.2 µm long smooth rod-shaped cells with a diameter of about 1.2±0.1 µm in 1 mM KNO_3_ solution, while 1.8±0.4 µm long cells with diameter of 0.8±0.2 µm are observed in 100 mM salt concentration (**Table 3**). In addition, root mean square (r.m.s) roughness of the E2152 mutant - as determined from analysis of the bacterial topography over a 500 nm × 500 nm surface area - decreases from 1.2±0.5 nm to 0.7±0.5 nm when increasing KNO_3_ salt concentration from 1 mM to 100 mM (**Table 4**). Qualitatively, a similar concentration-dependence of the r.m.s roughness and bacterial dimensions is found for E2146, E2302 and E2498 mutants (see details of data in **Tables 3** **-** **4**). Quantitatively, these results suggest a mean volume contraction (shrinkage rate) from 1 mM to 100 mM electrolyte concentration that is significantly larger for the bare E2152 strain (∼73%) as compared to that for other cell mutants (∼63%-65%). Furthermore, for a given ionic strength, r.m.s roughness for the strains exhibiting surface appendage at their outer periphery, is systematically larger than that for bare E2152 cells, result that is intuitively expected.

**Table 3:** Morphological characteristics of cells investigated in aqueous medium.

Strains	1 mM KNO_3_		100 mM KNO_3_		Average shrinkage
	Length	Diameter	Length	Diameter	
	(µm)	(µm)	(µm)	(µm)	(%)
2152	2.9±0.2	1.2±0.1	1.8±0.4	0.8±0.2	∼73%
2146	2.8±0.3	1.0±0.2	2.1±0.2	0.7±0.1	∼63%
2302	2.9±0.3	1.1±0.2	1.9±0.1	0.8±0.1	∼66%
2498	2.7±0.3	1.1±0.4	1.9±0.4	0.8±0.3	∼63%

Morphological characteristics (length and diameter) averaged over measurements carried out on 10 cells in 1 mM and 100 mM KNO3. The (average) extent to which cells shrink when passing from 1 mM to 100 mM solution is indicated.

**Table 4:** Mechanical characteristics of cells investigated in aqueous medium.

Strains	1 mM KNO_3_		100 mM KNO_3_	
	Non linear regime	Roughness	Non linear regime	Roughness
	(nm)	(nm)	(nm)	(nm)
2152	20-30	1.2±0.5	50-80	0.7±0.5
2146	80-120	1.5±0.6	250-350	1.1±0.8
2302	80-120	2.2±0.9	180-250	1.2±0.5
2498	30-50	1.7±0.5	80-120	±0.4

Mechanical characteristics (non linear regime) and average roughness carried out on 10 cells in 1 mM and 100 mM KNO3. The (average) extent to which cells shrink when passing from 1 mM to 100 mM solution is indicated. Maximal indentation in the non-linear regime (Hertzian behavior) and roughness values as obtained from AFM images of 500 nm × 500 nm bacterial surface area. Values are indicated under 1 mM and 100 mM KNO_3_ electrolyte concentration conditions.

### Nanomechanical properties of the bacterial strains

**Figure 4:**
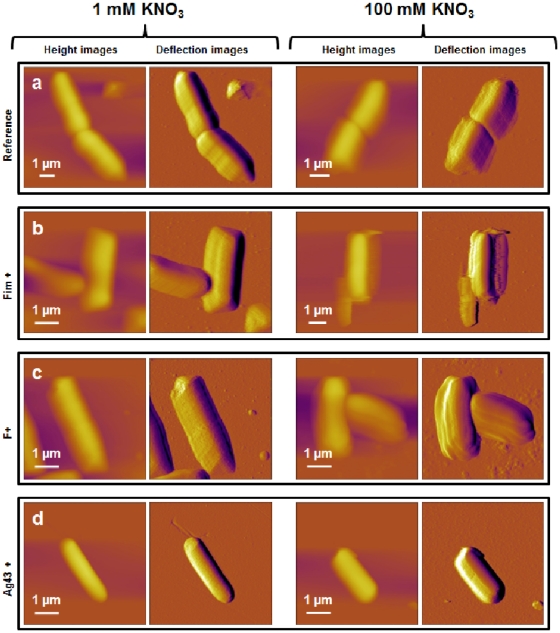
Nanomechanical properties of bacteria investigated in 1 mM KNO_3_. Deflection images (first column), elasticity maps (scale 0–20 MPa) (second column) and Young modulus distributions (third column) obtained in 1 mM KNO_3_ solution for a) E2152 b) E2146 c) E2302 d) E2498. Young modulus distributions were calculated within the spatial range marked by the white window positioned in the corresponding elasticity map (n = 256 force curves). Elastic (Young) moduli and bacterial spring constants were extracted from typical force-indentation curves shown in insets: open symbols are raw AFM data, solid lines stand for theoretical fits according to eq 5 (blue color, Hertzian non-linear regime) and straight lines represent fits on the basis of eq 4 (red color, linear or compliance regime associated to Turgor pressure contribution).

To determine the nanomechanical properties of the four bacterial strains of interest in this study, force-distance curves were recorded, converted into force-indentation curves, and subsequently analyzed according to eqs 4 and 5 for determining the bacterial spring constant, related to the inner Turgor pressure of the cell, and the Young modulus that reflects cell surface elasticity/rigidity. Also, use of 3D force volume mode rendered possible the generation of spatial distribution of Young Modulus (elasticity map) at the bacterial surface. **Fig 4** shows these elasticity maps for the four cells (a, b, c and d) in 1 mM KNO_3_ solution, together with the dispersion in Young modulus values as determined within the spatial region indicated by the white windows on the elasticity maps. Also, for the sake of illustration, typical force-indentation curves are given for each strain and were found to be in agreement with the force-indentation relationship expected from Sneddon model (eq 5), thereby rendering possible determination of Young moduli. Consistent with the work of Gaboriaud *et al*. [65], the force curves recorded on cell surfaces exhibit a non-linear regime at low loading forces (eq 5) followed by a linear regime for higher forces (eq 4). Bacterial spring constant values, *k*_cell_, were determined from the slopes of the linear portions of the force-*versus*-piezo displacement curves as described in Material & Methods section.

In **Fig 4** (1 mM salt concentration), the contrast in elasticity maps demonstrates that the cells were substantially softer than the PEI-coated glass slide for which a Young modulus of about tens of GPa is estimated. The curves recorded across the interphases formed between bacteria and aqueous medium yielded Young modulus of about 950 kPa for the reference E2152 bacteria, whereas for the other strains, elasticity was significantly lower with values of about 340, 500, and 700 kPa for E2146, E2302 and E2498, respectively (**Histogram S1a**). Elasticity maps recorded on the cells further showed a homogeneous contrast, indicating that curvature/edge effects did not influence substantially the shape of the force curves, as confirmed upon closer inspection of the dispersions in Young moduli values. The data further indicate that the presence of external polymeric structures obviously impacts the mechanical softness of the bacteria. Quantitatively, cell surfaces with type 1 *fimbriae* and F-*pili* are 3 to 2 times softer, respectively, as compared to that of E2152 cells devoid of these appendages. For the cell wall covered by Ag43 adhesins, elasticity is only 1.4 times softer than that determined for the reference strain. Additionally, the force-indentation curves clearly evidence that the range of indentation where non-linearity applies (eq 5), is depending on the presence and nature of bacterial surface appendages. For the reference bacteria, cell wall was non-linearly deformed over 20 to 30 nm while for E2146 and E2302 strains, this spatial range is about 80 to 120 nm. For E2498, the non-linear deformation spans over 30 to 50 nm. The bacterial spring constant *k*_cell_ of the various bacteria are further reported in **Histogram S1b**. In 1 mM KNO_3_ solution, *k*_cell_ is 0.18±0.06 N/m for the E2152 strain and for E2302, E2146 and E2498 cells, we obtain *k*_cell_ values of about 0.07, 0.08 and 0.11 N/m (see details in **Histogram** S**1b**), respectively. The here-determined magnitude of *k*_cell_ is in excellent agreement with that expected for gram-negative bacteria [38], [98]. The Turgor pressure of each bacterium was estimated from Arnoldi equations [86] (see further details in caption of **Histogram S1c**) taking for *k*_cell_ the values commented above and further assuming that value of *λ*, the stretching modulus of the bacterial envelope, is in the range 0.1 to 0.2 N/m as suggested in [85], [86], [99]. The corresponding procedure is detailed in **Fig S3**. The average bacterial radius required for the calculation was 500 nm. As a result, we obtained for the reference bacteria a Turgor pressure of 280–350 kPa, magnitude that is in agreement with previous results [100]. For bacteria exhibiting surface appendages, the Turgor pressure is found to be significantly lower with values in the range 80–120 kPa for bacteria with type 1 *fimbriae* and F-*pili*, and 150–200 kPa for bacteria covered by Ag43 adhesins. These rough estimations point out the role played by cell wall external structures for improving the stability of bacterial envelope against osmotic stress.

**Figure 5:**
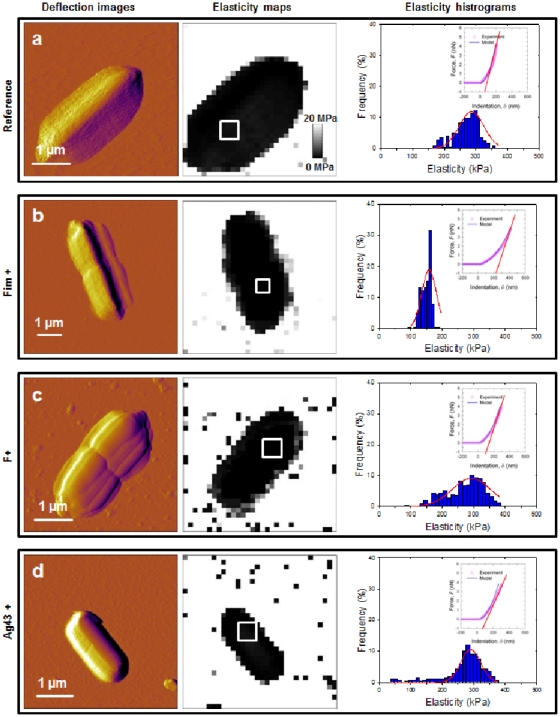
Nanomechanical properties of bacteria investigated in 100 mM KNO_3_. Deflection images (first column), elasticity maps (scale 0–20 MPa) (second column) and Young modulus distributions (third column) obtained in 100 mM KNO_3_ solution for a) E2152 b) E2146 c) E2302 d) E2498. Young modulus distributions were calculated within the spatial range marked by the white window positioned in the corresponding elasticity map (n = 256 force curves). Elastic (Young) moduli and bacterial spring constants were extracted from typical force-indentation curves shown in insets: open symbols are raw AFM data, solid lines stand for theoretical fits according to eq 5 (blue color, Hertzian non-linear regime) and straight lines represent fits on the basis of eq 4 (red color, linear or compliance regime associated to Turgor pressure contribution).

In **Fig 5**, mechanical measurements are shown under conditions where KNO_3_ concentration is 100 mM. Young moduli of about 300 kPa were obtained for the E2152 reference bacteria and we found moduli of 152, 320 and 298 kPa for E2146, E2302 and E2498, respectively (**Histogram S1a**). In addition, the deformation is non-linear over 50 to 80 nm for the E2152 reference bacteria, while this characteristic distance of deformation is found to be significantly more important for E2146, E2302 and E2498 (350, 250 and 120 nm, respectively, see **Table 4**). At 100 mM salt concentration, no significant change in *k*_cell_ value was found for the four bacterial strains examined (0.003 N/m, **Histogram S1b**), and the evaluation for the corresponding Turgor pressure leads to values in the range 20 to 40 kPa.

### Electrokinetics

**Figure 6:**
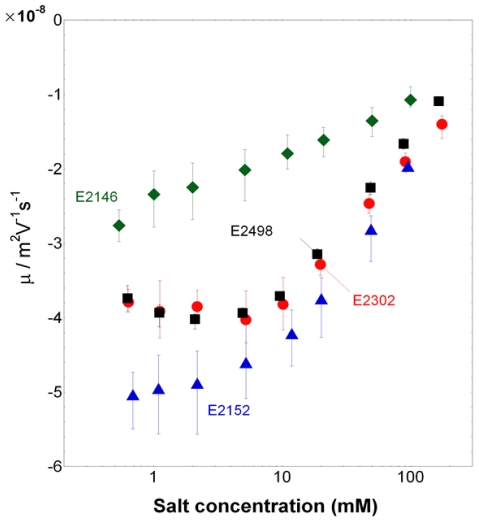
Electrophoretic mobility of the bacteria. Electrophoretic mobility *versus* KNO_3_ salt concentration as measured for E2152, E2146, E2302, and E2498 strains (indicated). Solution pH∼6.6.

The dependence of the electrophoretic mobility *µ* on ionic strength is reported for the bacterial strains E2152, E2146, E2302 and E2498 in **Fig 6**. For all strains, *µ* is negative, as expected for biological particles [70], and decreases in absolute value upon increase of salt concentration, in agreement with enhanced screening of the charges embedded within the cell wall and/or surface appendage by ions present in the medium. The electrokinetic behavior of the cells is further typical of that for soft particles, as judged from the presence of an asymptotic plateau value for the electrophoretic mobility at sufficiently high ionic strengths [66], particularly evident when patterns depicted in **Fig 6** are plotted according to a linear scale in ionic strength. Additionally, similarly to the nanomechanical features of bacteria reported above, the presence/absence and nature of the surface appendage impact notably the electrokinetic properties of the cells.

**Figure 7:**
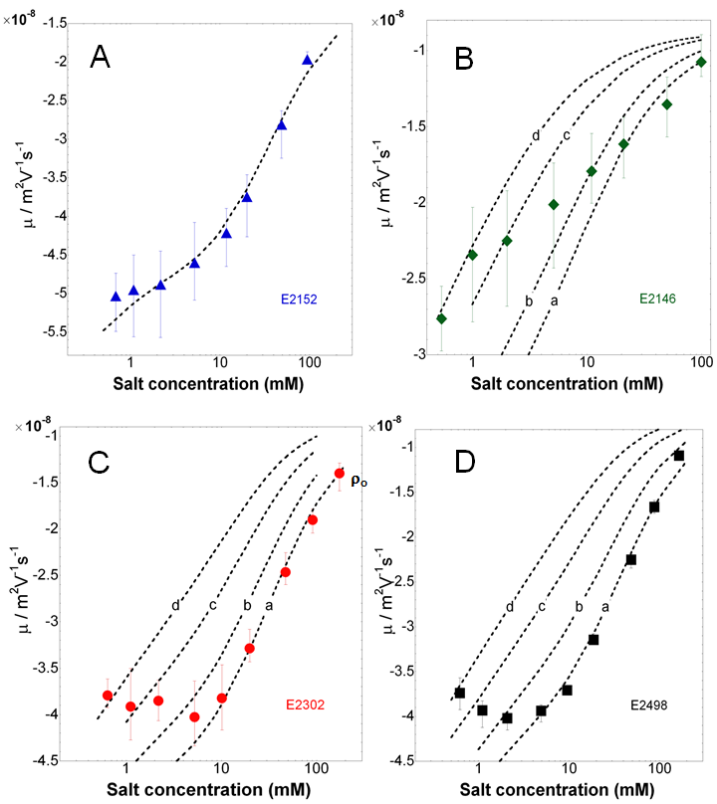
Electrokinetic analysis of the bacterial electrophoretic mobilities. Quantitative analysis of the electrokinetic patterns of (A) E2152, (B) E2146, (C) E2302, (D) E2498. The charge density 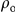, the characteristic flow penetration length 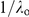, the heterogeneity length scale 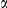 and the thickness *d* of the soft bacterial component are indicated below. (A) E2152: 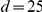
nm. 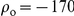
mM, 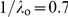
nm, 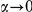. (B) E2146: 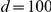
nm. 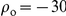
mM, 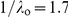
nm, (curve a) 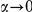, (curve b) 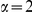
nm, (curve c) 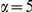
nm, (curve d) 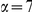
nm. (C) E2302: 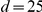
nm. 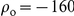
mM, 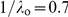
nm, (curve a) 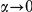, (curve b) 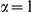
nm, (curve c) 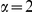
nm, (curve d) 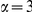
nm. (D) E2498: 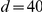
nm. 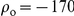
mM, 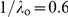
nm, (curve a) 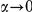, (curve b) 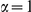
nm, (curve c) 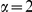
nm, (curve d) 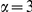
nm. N.B.: Values for 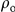 and 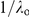 are indicated with a precision of ±10% in relation with error bars of experimental data.

In details, three types of behavior may be distinguished: that of the reference cell E2152, for which |*µ*| and its increase with decreasing ionic strength is most significant; that of E2146 strain for which |*µ*| is lowest at fixed concentration and its increase when lowering electrolyte concentration is least pronounced; and finally an intermediate behavior which is that of the strains E2302 and E2498 for which |*µ*| significantly increases when decreasing ionic strength from 100 mM to about 10 mM, and subsequently levels off for concentrations lower than 10 mM. Analysis of the data was carried out according to a methodology detailed elsewhere [65] on the basis of the theory outlined in previous section. For that purpose, the length scale associated to the thickness of surface appendage and/or cell wall was estimated from nanomechanical AFM analysis of the bacteria in 1 mM KNO_3_ solution, *i.e.* from the spatial range where non-linearity between loading force and nano-indentation applies, thus providing *d*∼25 nm for E2152, *d*∼40 nm for E2498 and *d*∼100 nm for E2146 and E2302. It is indeed under such low ionic strength conditions that deformation of the very bacterial soft component upon compression/indentation by the AFM tip is least impeded by a possible concomitant deformation of bulk cytoplasm [65]. This important element, further commented in the Discussion section, is supported by the large Turgor pressure of the cells obtained in 1 mM solution as compared to that determined in 100 mM salt concentration, in line with results and observations by Gaboriaud et *al.* [65]. Analysis of the data are given in **Fig 7** for each strain, recalling that the electrokinetic parameters *ρ*_0_ (expressed below as a volume concentration of charges) and *λ*_0_ are jointly determining the slope and magnitude of the mobility as a function of ionic strength in the high salt concentration regime, while interphasial heterogeneity of the bacterium (parameter *α*) may further come into play at sufficiently low ionic strengths as a result of possible swelling of the cell wall/surface appendage. The reader is referred to [68] for further details. The legend of **Fig 7** collects the magnitude of the electrokinetic parameters obtained for the various bacteria, having in mind that values are given with a precision of±10%, in relation with the error bars of experimental data. Remarkably, data for the reference bacteria E2152 could be reproduced by theory over the whole range of ionic strength examined in this study. The charge density for this strain is high (*ρ*_0_ = −170 mM) as compared to that obtained for other gram-negative bacteria like *Shewanella* [65] and is in good agreement with that determined by Sanohara et *al.* for *E. coli* [101]. The analysis suggests that it is not necessary to introduce any interphasial heterogeneity of the bacteria for adequately reproducing electrokinetic behavior at low ionic strengths. The rate of increase of |*µ*| upon decrease of ionic strength may be indeed solely attributed to a significant polarization of the electric double layer around/within the bacteria, as expected for such large values of charge density [68]. Also, an hydrodynamic penetration length 1/λ_0_ of about 0.7 nm is determined, which denotes a poor intrusion of the electroosmotic flow within the thin cell wall that serves as only soft component for the reference strain devoid of type 1 *fimbriae*, F-*pili* or Ag43 protein. For bacteria with type 1 *fimbriae* (E2146), we obtain significantly lower charge density (ρ_0_ = −30 mM) and larger hydrodynamic penetration length (1/λ_0_ = 1.7 nm) than for the E2152 strain. This indicates that the charges responsible for the motion of the particles upon action of the electric field are either present in lower number or distributed over larger volume than for E2152 cells and that the supporting soft structure is significantly permeable, *i.e.* inhibits flow penetration to a lower extent than within the cell wall surrounding the reference bacteria E2152. Additionally, mobility values for ionic strengths lower than ∼20 mM are compatible with an increase of *α*, *i.e.* a heterogeneous extension of the bacterial appendage by electrostatically-driven swelling processes connected to repulsive interactions between neighboring charges carried by the fimbriae. For the E2498 strain covered by Ag43 protein layer, electrokinetic data are excellently reproduced in the ionic strength range 5 mM to 100 mM adopting the values *ρ*_0_ = −170 mM and 1/λ_0_ = 0.6 nm. For lower ionic strengths, the slight decrease in |*µ*| with decreasing ionic strengths is interpreted by a swelling of the outer edge of the protein layer, as judged by the increase in length scale 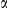 that allows the recovering of data points collected at KNO_3_ concentrations lower than 5 mM. Similarly to E2152, the set of electrokinetic parameters (*ρ*_0_, *λ*_0_) obtained for E2498 typically pertains to a soft structure that is rather compact, highly charged, and poorly permeable. Finally, despite the proven presence of F-*pili* at the surface of E2302 strain, the dependence and magnitude of |*µ*| with ionic strength depicts features that are surprisingly similar to those obtained for the strain E2498. It was systematically verified that an analysis taking into account a thickness *d*∼100 nm for these F-*pili* rendered impossible any appropriate fit of the data at large ionic strengths and further systematically required a charge density and hydrodynamic penetration length of magnitudes *ρ*_0_∼−160 mM, 1/λ_0_∼0.6 nm that mark the presence of compact soft structures (see values derived for E2152 and E2498) rather than that of the expected long, flexible filaments (see values obtained for E2146). An explanation for this counterintuitive result is inferred upon closer inspection of the AFM images detailed in **Fig 3**. Indeed, in the case of E2146, type 1 *fimbriae* clearly constitute a continuous polymeric layer surrounding the cell membrane whereas for E2302, the number of observed F-*pili* is extremely low (to a maximum of 5) and by no means may be assimilated to a polymeric layer of which electrohydrodynamic properties can be tackled on the basis of a mean field model. In other words, the quantities *ρ*_0_ and 1/*λ*_0_ derived for E2302 suggest that the electrokinetic properties for these bacteria are mainly governed by the cell membrane supporting the F-*pili* rather than by the F-*pili* themselves which are either too scarce for generating any predominant contribution to the overall mobility of the bacteria, or completely retracted along the cell wall, thereby covering heterogeneously a insignificant spatial fraction of the overall bacterial surface. This is supported by the excellent quantitative recovering of the electrokinetic data for E2302 at ionic strengths larger than 10 mM, taking for the cell surface appendage thickness the value *d*∼25 nm, *i.e.* that associated to the cell wall thickness for the reference strain E2152. For salt concentration below 10 mM, data measured for E2302 are further in line with a heterogeneous extension of the bacterial soft interphase, which is probably associated to a stretching of the few F-*pili* surrounding the cell and/or that of the supporting cell membrane.

For the strain E2146, the electrokinetic analysis points out a value of the dimensionless parameter 1/*λ*_0_ which is about 50 times lower than the typical thickness ∼100 nm of the fimbriae layer. Additionally, within the salt concentration range of interest in this study, the fimbriae layer thickness is much larger than the Debye layer thickness, classically denoted as *κ* ^−1^. These elements indicate that for the E2146 strain, the electroosmotic flow solely probes the charges located at the outer edge of the soft component of the bacteria with as a result an insignificant contribution of charges located within the supporting cell wall. Such reasoning is in line with the observation by Duval et *al.* [102] on the electrokinetic features of fibrillated *S. Salivarius* of which electrophoretic mobility is determined by the outer corona of the fibrils only, and is further supported by numerical simulations performed according to the electrokinetic formalism for soft multi-layered bioparticles adapted for cylindrical geometry [79] (not shown) (and see review in [70]). For the strain E2498, the electrohydrodynamic analysis discussed above was carried out taking an overall soft layer thickness of ∼40 nm as inferred from AFM mechanical results. This layer however encompasses not only the Ag43 protein layer but also the cell wall which is about 25 nm thick, as judged from AFM results obtained for the reference strain E2152. Consequently, the thickness for the only Ag43 protein layer is about 15 nm, in good agreement with values reported in the literature [70]. The important feature is that the typical dimension of this layer is of the order of *κ* ^−1^ in 1 mM salt concentration. As a result, ignoring the two layer-structure for the soft component of E2498 and assimilating it to a single component within electrokinetic analysis, may lead to erroneous estimation of the local electrostatic potential distribution all across the soft interphase, and thus misinterpretation of the electrophoretic mobility. In view of the separate determination of the thicknesses of cell wall and Ag43 protein layer and further recalling that electrokinetic features of the only cell wall are known from analysis of mobility data collected for E2152, we therefore refined our interpretation of results depicted in **Fig** **7D** for E2498 by amending the electrokinetic formalism for soft multi-layered bioparticles developed in spherical geometry [79] to the case of cylindrical particles. Along this line, the soft component of E2498 now consists of two layers, the cell wall of known thickness and charge density (*ρ*_0_ = −170 mM), and the 15 nm thick Ag43 protein layer for which *ρ*_0_ and flow penetration degree 1/*λ*_0_ are to be determined. Corresponding results are essentially identical to those obtained by assimilating the two-layer structure of E2498 to a single shell basically because the charge density as retrieved for the Ag43 protein layer is fortuitously of the same order of magnitude than that derived for the cell wall only (based on analysis of E2152).

## Discussion

### On the relationships between nature of bacterial external structures, their mechanical elasticity and electrohydrodynamic properties

In this work, the electrokinetic and elastic properties of *E. coli* interphases are determined by electrokinetic and AFM nanomechanical analyses. The investigation is carried out for four bacterial strains that differ according to the presence/absence and nature of surface appendages as detailed in **Table 1**.

For sufficiently large electrolyte concentrations, the repulsive electrostatic interactions between neighboring charged sites within the soft parts of the bacteria are screened. Attractive segment-segment interactions therefore lead to collapse of the associated gel-like structure of the appendage/cell wall surrounding the bacteria. Under these conditions, the electrokinetic signature of all bacteria can be satisfactorily interpreted by considering the soft surface layer as homogeneous (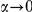). More in details, electrophoretic mobility measured for the reference bacteria E2152 are quantitatively explained taking a charge density *ρ*_0_∼−170 mM and a hydrodynamic penetration length 1/*λ*_0_∼0.7 nm. These values reflect the electrohydrodynamic features of the cell wall which is further characterized by an important Young Modulus (950 kPa). This extreme mechanical rigidity of the cell wall, as compared to that of other appendages carried by the mutant bacteria, agrees with the absence of swelling for the cell wall over the whole range of ionic strengths 0.5–100 mM examined by electrokinetics. Indeed, the degree of swelling for a given charged polymeric layer is subject at equilibrium to the condition of zero net osmotic pressure Π = 0, where Π contains an entropic contribution, Π_mix_, related to polymer-solvent mixing, an electrostatic contribution Π_elec_ determined by the charge density *ρ*_0_, and an elastic contribution Π_elastic_ which is intrinsically governed by the rigidity of the polymer chains. For E2152, the rigidity of the cell wall prevents from significant swelling despite the large volume charge density determined for the soft part of this bacterium. Additionally, the compact and rigid character of the cell wall is supported by a sub-nanometric electroosmotic flow penetration as subsumed in the quantity 1/*λ*_0_. For the sake of comparison, values as large as 1/λ_0_∼2–4 nm were obtained for *Shewanella* bacterial strains [65] or other highly permeable bioparticles like viruses [79]. In case of E2498 strain with the Ag43 protein layer at the surface, swelling takes place for salt concentrations below∼5 mM, as indicated by the corresponding increase of the parameter *α*. Because the charge density obtained for E2498 is of the same order of magnitude than that determined for the reference strain, this result suggests that the Ag43 protein layer is more flexible, less rigid than the cell wall component, thus allowing for layer extension as driven by the repulsive interactions between neighboring charged chains. This is confirmed by AFM analysis of the mechanical properties of E2498, which indicates a Young modulus that is about 30% lower than that for E2152. The electrokinetic and mechanical features of the mutant bacteria E2146 covered by the type 1 *fimbriae* considerably differ from those obtained for E2152 and E2498. The charge density and Young modulus obtained for the E2146 strain are indeed ∼6 and ∼2–3 times lower as compared to values collected for E2152 and E2498, while the typical flow penetration length is about ∼2.5 times larger. These comparisons clearly indicate the loose structure of the fimbriae layer, its resulting larger permeability with, in turn, a significant layer swelling at salt concentrations below ∼20 mM. Finally, the electrophoretic features of E2302 resemble those of E2498 and E2152 at least in the high ionic strength regime, mainly because the presence of few F-*pili* and their discontinuous positioning at the bacterial surface, as imaged by AFM, do not lead to a significant contribution to the overall mobility of the bacterium which is instead determined by the electrohydrodynamic properties of the supporting cell wall. However, the nanomechanical softness of E2302 bacterial interphase seems to be mainly controlled by the very loose F-*pili*, even if those are present at a very low density. This is inferred from the low value of the Young modulus (∼500 kPa) obtained for this strain, which is about 50% lower than that determined for the reference E2152 strain. Such sensitivity of the AFM mechanical analysis to the presence of a few surface appendages only is explained by the low spring constant chosen for the AFM tip, thus allowing for refined local measurements over a few nm^2^ spatial resolution while electrophoresis should be rather considered as a technique yielding averaged information over the whole surface area of the bacteria.

### On the response of *E. coli* bacteria on osmotic stress, effects on cell spring constant (or equivalently inner Turgor pressure)

The AFM and electrokinetic data discussed above demonstrate the key roles played by mechanical rigidity and volume charge density of cell wall and surface appendages in determining their propensity to swell upon lowering ionic strength. With regard to the inner Turgor pressure of the cells that is accessible by AFM only, we found that under 1 mM salt concentration condition, it decreases appreciably with the presence of external polymeric filaments like type 1 *fimbriae* and F-*pili*. On the contrary, in 100 mM salt concentration, regardless of the presence and nature of surface appendage, the Turgor pressure remains roughly identical for all strains and is systematically lower in magnitude than that determined in the low ionic strength regime. It is known that bacteria respond to changes in the osmolarity of their environment by regulating their Turgor pressure through exoosmotic water release [103]. Our results show that the presence of loose, flexible surface appendage around the bacteria under low electrolyte concentration condition acts as a protective barrier, thereby attenuating the impact of changes in extracellular ionic strength and lowering the osmotic pressure constraint. The underlying processes are possibly related to the building up of chemical and ionic gradients within the (swollen) appendage associated-gel layer, with as a result a significant suppression of the osmotic stress exerted on the cytoplasm, thereby lowering the inner Turgor pressure. Another explanation for the dependence of the Turgor pressure on surface appendages is quantitatively provided in **Figs S3–S4** where the reader is referred for details. Basically, the analysis suggests that there is a quantitative correlation between the stretching modulus of the bacterial envelope, *λ*, and the presence/absence and nature of surface appendage anchored at the cell wall. In details, it is found that *λ* significantly decreases with the presence of long/flexible appendages (type 1 *fimbriae* and F-*pili*), meaning that these tend to decrease the surface energy of the overall bacterial envelope. Recalling that the Turgor pressure is intrinsically depending on the stretching modulus *λ* of the bacterial envelope and the cell spring constant (3), the AFM study carried out on the four bacterial strains indicates that the dependence of the Turgor pressure on bacterial surface structures may be accounted for upon arguing the impact of these more or less loose structures on the quantity *λ* or equivalently on the bacterial surface energy.

In 100 mM salt concentration, where stretching of the surface appendage is less marked and/or insignificant, the range of the non-linear regime in the force indentation curves, supposedly associated to the deformation of the surface appendage, is surprisingly broader than that obtained in 1 mM concentration. As invoked in preceding sections, this feature is attributed to the very low Turgor pressure of the cells as a result of water release, and the high osmotic stress bacteria are facing is not regulated by the presence of any surface appendage as confirmed by the quasi-identical Young moduli obtained for the four bacterial strains examined here (**Histogram S1a**). The picture is basically that of significantly contracted bacteria (which is supported by the study of bacterial morphology, see **Table 1**) due to partial loss of inner water so that the interphase with surrounding medium is rendered very elastic or flexible, as confirmed by the low values of Young moduli (**Histogram S1a**). Consequently, upon compression of the AFM tip onto the bacteria, the deformation concerns not only the bacterial soft component but also the elastic cytoplasm volume. This leads to a misleading interpretation when attributing the non-linear mechanical behaviour to the compression of the surface appendage and/or cell wall only.

### Conclusion

In this paper, we report a systematic analysis of the nanomechanical properties (Young modulus, inner Turgor pressure) and electrokinetic features (electroosmotic flow penetration length scale and volume charge density) for four *E. coli* bacterial strains expressing or not the surface appendages type 1 *fimbriae*, type-F *pili* or Ag43 protein layer. It is found that bacterial elasticity and inner Turgor pressure strongly decrease in the presence of loose surface filaments under conditions of low medium ionic strength (1 mM) while these properties remain roughly identical in 100 mM salt concentration where exoosmotic water loss and bacterial contraction are most significant. A qualitative connection is made between the rigidity of the bacterial interphase and their swelling features upon lowering ionic strength, thereby pointing out the concomitant importance of the charge density they carry together with their intrinsic elasticity in governing the degree of interphase extension. It is further shown how the electrophoretic patterns (mobility *versus* ionic strength) are correlated to the presence/absence, nature of the surface appendages carried by the bacteria, in excellent agreement with the expected genotypes and phenotypes of the bacteria and their imaging by AFM.

In forthcoming studies, the here-reported static analysis of the electrokinetic and nanomechanical properties of the four *E. coli* bacterial strains will be extended by a dynamic investigation where bacteria will be subjected to electric and mechanical frequency-dependent stimulations. It is believed that the complete set of physico-chemical data collected for these bacteria with known interphasial structures, will then allow for a quantitative understanding of their adhesion features onto substrates with well-controlled surface chemistry.

## Supporting Information

Figure S1 **Force curves recorded for the strain E2152 (4 graphs on the left) and for the strain E2302 (4 graphs on the right) with experimental data marked by the open symbols (pink), Hertz expression by the solid blue curve and exponential regression by the solid red line at 1 mM (panels A and C) and 100 mM (panels B and D) KNO3 concentration.** The plots depicted in panels C and D are zooms of the data points within the onset of the non-linear regime as materialized by the windows given in panels A and B.
(EPS)

Figure S2 **Force curves recorded for the strain E2146 (4 graphs on the left) and for the strain E2498 (4 graphs on the right) with experimental data marked by the open symbols (pink), Hertz expression by the solid blue curve and exponential regression by the solid red line at 1 mM (panels A and C) and 100 mM (panels B and D) KNO3 concentration.** The plots depicted in panels C and D are zooms of the data points within the onset of the non-linear regime as materialized by the windows given in panels A and B.
(EPS)

Figure S3 **Bacterial spring constant as a function of Turgor pressure for stretching moduli of the bacterial envelope fixed at values of 0.10, 0.15, 0.20, 0.30 and 0.40 N/m.** The dashed horizontal line corresponds to the kcell value obtained by AFM for the E2152 strain in 1 mM KNO3 electrolyte concentration.
(EPS)

Figure S4 **Bacterial spring constant in 1 mM KNO3 electrolyte concentration as a function of Turgor pressure.** The curves correspond to computations performed with the minimum stretching moduli (indicated) λmin which allow retrieving of the experimentally determined k_cell_ for E2152, E2146, E2302 and E2498 bacteria.
(EPS)

Table S1 **Phenotypic assays to validate the constructed strains.**
(DOCX)

Histogram S1
**Variations of mechanical properties for each bacterial structures.** a) Elastic modulus (or Young modulus) in 1 mM and 100 mM KNO_3_ solution. b) Bacterial spring constant in 1 mM and 100 mM KNO_3_ solution. c) Turgor pressure in 1 mM and 100 mM KNO_3_ solution. ^†^ Elasticity and bacterial spring constant are averages calculated from 1024 force curve measurements. ^‡^ Turgor pressure estimations were done according to the equation 
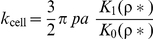
where *p* is the Turgor pressure, *a* the bacterium radius (∼500 nm), K_0_ and K_1_ are modified Bessel functions of the second type of order 0 and 1, respectively. ρ* stands for the reduced curvature radius defined by 
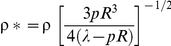
with ρ the cantilever tip radius (ρ∼20 nm) and λ the stretching modulus of the bacterial envelope (0.1≤λ ≤0.2 N/m). See Test S1 for further details.(EPS)

Histogram S2 **Histograms of elasticity of bacterial cell envelops determined in 1** **mM KNO3 solution according to Hertz model only (black) and according to Hertz model after suppression of the electrostatic part of the force versus separation distance curve (grey).**
(EPS)

Text S1 (DOC)
