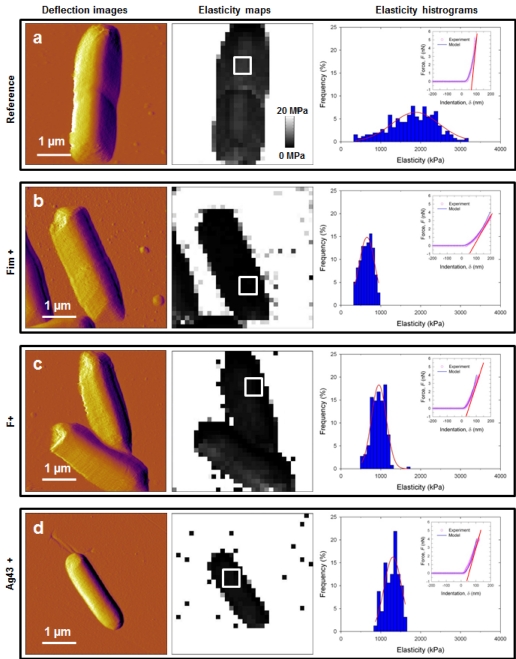# Correction: Bacterial Surface Appendages Strongly Impact Nanomechanical and Electrokinetic Properties of *Escherichia coli* Cells Subjected to Osmotic Stress

**DOI:** 10.1371/annotation/2d1cbf5c-d0f0-40c6-8f32-eb034182538c

**Published:** 2011-07-25

**Authors:** Grégory Francius, Pavel Polyakov, Jenny Merlin, Yumiko Abe, Jean-Marc Ghigo, Christophe Merlin, Christophe Beloin, Jérôme F. L. Duval

Figure 4 was erroneously uploaded as a duplicate of Figure 3. The correct Figure 4 can be viewed here: 

**Figure:**