# Supplementary material for: Bacterial Surface Appendages Strongly Impact Nanomechanical and Electrokinetic Properties of Escherichia coli Cells Subjected to Osmotic Stress
Source: PLoS One. 2011 May 31;6(5):e20066. doi: 10.1371/journal.pone.0020066 (PMC3105017; doi:10.1371/journal.pone.0020066)
Supplement: Table S1 — Phenotypic assays to validate the constructed strains. (DOCX) [file pone.0020066.s005.docx]

**Table S1.** Phenotypic assays to validate the constructed strains.

| **Strains** | **Motility** | **Yeast Agglutination** | **Biofilm formation** | **Bacterial Autoaggregation** | **Ag43 immunodetection** | **M13 phage sensitivity** |
| --- | --- | --- | --- | --- | --- | --- |
| **E2152** | - | - | +/- | - | - | - |
| **E2146** | - | +++ | +++ | - | - | - |
| **E2498** | - | - | ++ | +++ | +++ | - |
| **E2302** | - | - | +++ | +/- | - | +++ |

For all mutant strains of interest in this study, the results of motility, yeast agglutination and biofilm formation tests are reported for type 1 fimbriae, western blot immunodetection and bacterial autoaggregation for Ag43 adhesin, and M13 phage sensitivity and biofilm formation for F pili.
