# Supplementary material for: Bacterial Surface Appendages Strongly Impact Nanomechanical and Electrokinetic Properties of Escherichia coli Cells Subjected to Osmotic Stress
Source: PLoS One. 2011 May 31;6(5):e20066. doi: 10.1371/journal.pone.0020066 (PMC3105017; doi:10.1371/journal.pone.0020066)
Supplement: Text S1 — (DOC) [file pone.0020066.s008.doc]

## **Supporting Information S1**

**On the impact of the electrostatic part of the force curves on the determination of nanomechanical parameters.**

Force measurements were recorded in 1 mM and 100 mM KNO_3_ concentrations for the four bacterial strains E2152, E2302, E2146 and E2498 considered in the study. Typical data are given in Figures S1-S2 where the quality of the data fit according to Hertz expression within the non-linear regime of the force curves is highlighted. Additionally, an exponential regression of the data is indicated as an indicator of the role played by electrostatic forces at the onset of the non-linear regime. As judged from the appropriateness of the Hertz expression in reproducing data for very low loading forces (0.1-0.3 nN), the results suggest that electrostatic forces are most operative under low ionic strength condition (1 mM KNO_3_ concentration) where screening of the volume charge carried by the bacterial envelop is least screened by ions present in the medium. In addition, it is clear that electrostatic forces are most significant for the E2152 and E2498 strains, *i.e.* those that exhibit the larger volume charge densities ρ_0_ as determined independently from electrokinetic analysis. This qualitative result is in line with expectation.

A rigorous determination of the nanomechanical parameters of the bacteria requires excluding from the mechanical analysis of the non-linear regime of the force curves the spatial range where electric double layer repulsion between AFM tip and bacteria is present. This is done upon shifting accordingly the contact point. When doing so, it appears that this shift is negligible as compared to the overall piezo-drive and does not affect to a significant degree the general pattern of the modeled force versus indentation curve. This is further verified upon computation of Young moduli when taking into account the aforementioned shift of the contact point between bacteria and AFM tip as a result of the presence of electrostatic forces. The results indicate that a slight under-estimation of the elastic modulus of about 1 to 5% (according to the strain) is obtained if including in the mechanical analysis data points located within the electrostatic regime of the force versus separation distance curves (Histogram S2).

As an example, we obtained for E2152 strain elastic moduli of 953 kPa and 981 kPa when reasoning on the basis of Hertz model without and with suppression of the part of the force curves reflecting electrostatic double layer repulsion, respectively. The corresponding difference in elastic modulus is for that strain about 3%.

**Evaluation of inner bacterial turgor pressure.**

Turgor pressure estimations were done using the implicit equation $k_{\text{cell}}=\frac{3}{2}\pi\text{ }pa\text{ }\frac{ \mathrm{K}_{1}(\text{ρ*})}{ \mathrm{K}_{0}(\text{ρ*})}$ where *p* is the Turgor pressure, *a* the bacterium radius (~500 nm), K_0_ and K_1_ are Bessel functions and ρ* the reduced curvature radius defined by $\text{ρ}*=\text{ρ }\left[\frac{ 3pR^{3}}{ 4\left(\lambda-pR\right)}\right]^{-1/2}$with ρ the cantilever tip radius (ρ ~ 20 nm) and λ the stretching modulus of the bacterial envelope (0.10 ≤ λ ≤0.2-0.40 N/m). We have to express the bacterial spring constant (which is obtained from AFM data analysis of the compliance regime) in the form of a parametric function of the turgor pressure

$k_{cell}\left(p,\lambda\right)\approx p\cdot\mathrm{f}\left(p,\lambda\right)$ (1)

, with $\mathrm{f}\left(p,\lambda\right)=\frac{\text{K}_{1}(p,\lambda)}{\text{K}_{0}(p,\lambda)}$ where *p* is the searched parameter. To evaluate *p*, we fixed the stretching modulus λ at values of 0.10, 0.20, 0.30 and 0.40 N/m which are in agreement with typical magnitudes reported in literature (Arnoldi *et al.* 2000 and Boulebitch 2000, complete references are given in the main text).

For low bacterial spring constants *k*_cell_, the stretching modulus poorly impacts the Turgor pressure, *p*, whereas for larger *k*_cell_, the stretching modulus λ significantly governs the magnitude of *p* (Figure S3).

On the basis of the parametric curves plotted in Figure S3, it is possible to obtain the Turgor pressure of the bacterial strains of interest in the study recalling that the *k*_cell_ values are known from analysis of the compliance region in the force *versus* indentation curves. For the sake of illustration, the case of the E2152 strain in 1 mM KNO_3_ concentration is treated in Figure S3. It is clear that the parametric curves computed for realistic values of λ adequately reproduce the experimentally determined *k*_cell_ for λ > 0.15 N/m. More precise computations indicate that the minimum value required for reproducing *k*_cell_ of E2152 strain in 1 mM KNO_3_ electrolyte solution is λ = 0.18 N/m. Assuming that the quantity λ is indicative of the surface energy of the bacterial envelope, we may postulate on the basis of thermodynamic arguments that a good approximation for λ corresponds to the minimum value (*e.g.* minimum bacterial surface energy) that allows for retrieving the measured bacterial spring constant. Following such a strategy, it is then possible to estimate λ for the four bacterial strains E2152, E2498, E2302 and E2146. This is illustrated in Figure S4 where the parametric curves in agreement with the *k*_cell_ values of each strain are depicted. The corresponding (minimum) values of λ are also indicated.

From Figure S4, the dependence of the quantity λ on the type of strain is evidenced. This dependence suggests that λ decreases for bacterial envelopes covered with polymeric appendages (strains E2146 and E2302), meaning that the presence of such flexible surface appendages tends to decrease the surface energy of the bacterial envelope.

**Bacterial strains**

The four strains analyzed in this study are all isogenic strains and have been specifically created for the purpose of the analysis. All mutations or insertions present in these strains come from either already existing and previously characterized strains, or correspond to deletion of genes, which deletion by other antibiotic markers are known to lead to the expected phenotypes. A precise list of strains (origin and construction) is provided in Table 1 in the main text. In addition, the classical phenotypes associated with deletion of the constitutive expression of the three different adhesins studied here (*i.e.* type 1 fimbriae, the Ag43 adhesin and the F pili) were systematically verified by different assays such as yeast agglutination and biofilm formation for type 1 fimbriae, western blot immunodetection and bacterial autoaggregation for the Ag43 adhesin, and M13 phage sensitivity and biofilm formation for the F pili (see Table S1 for details).
